# Research on Digital Steganography and Image Synthesis Model Based on Improved Wavelet Neural Network

**DOI:** 10.1155/2022/7145387

**Published:** 2022-06-01

**Authors:** Xujie Li, Rujing Yao, Jonghan Lee

**Affiliations:** ^1^Department of Art, Tianjin Renai College, Tianjin 301636, China; ^2^Department of Formative Convergence Arts, Hoseo University, Asan 31499, Republic of Korea; ^3^Academy of Art and Design, Anhui University of Technology, Ma' anshan, Anhui 243002, China

## Abstract

Network compression coding technology is a research hotspot in the field of digital steganography and image synthesis. How to improve image quality while achieving short compression time is a problem currently faced. Based on the improved wavelet neural network theory, this paper constructs a digital steganography and image synthesis model. The model first tracks the contour of the digit to be recognized, then equalizes and resamples the contour to make it translation-invariant and scaling-invariant, and then uses multi-wavelet neural network clusters to stretch the contour shell to obtain orders of magnitude multi-resolution and its average, and finally, these shell coefficients are fed into a feedforward neural network cluster to identify this handwritten digit, solving the problem of multi-resolution decomposition of contour shells while having a high sampling rate. In the simulation process, the classification model that a single pixel is a text/non-text pixel is trained on the original gray value of the gray pixel and its neighboring pixels, and the classified text pixels are connected to a text area through an adaptive MeanShift method. The experimental results show that it is feasible to use multi-wavelet features for handwritten digit recognition. The model combines the neural network and the genetic algorithm, making full use of the advantages of both, so that the new algorithm has the learning ability and robustness of the neural network. The compression ratio after compression by ordinary wavelet coding, PSNR, MSE, and compression time are 8.4, 25 dB, 210, and 7 s, respectively. The values are 11.7, 24 dB, 207, and 11 s, respectively. At the same time, the peak signal-to-noise ratio is higher and the mean square error is lower, that is, the compression quality is better, and the accuracy of digital steganography and image synthesis is effectively improved.

## 1. Introduction

The main reason why images can be compressed is that there is redundant data in images. In today's big data era, in order to reduce the pressure of mass data storage and transmission, it is necessary to compress mass data, that is, to reduce redundancy in data [1–3]. Redundancy comes from the correlation between data. Removing redundant data means reducing some of the redundancy in the data without causing people to misunderstand the transmitted data. People have carried out a lot of research and statistics on the visual effects of traditional image data and found that there is a large correlation between adjacent lines and adjacent frames of the image [4–6]. Inter-pixel redundancy is the data redundancy generated by the correlation between pixels. For static pictures, there is spatial redundancy. The visual contribution of many single pixels in a single picture to the entire image is redundant. Neighboring pixel gray values are used for inference. For dynamic pictures or videos, most of the adjacent corresponding point pixels are gradually passed through slowly, and there will also be temporal redundancy (inter-frame redundancy).

As a representative of emerging mathematical analysis, wavelet analysis has good time-frequency localization ability, and can be better reflected in human vision, so it is widely used in image compression. In order to obtain higher compression ratio and compression quality and have the advantages of wavelet in the compression process, wavelet transform is usually combined with other compression codes [7–11]. Neural network is also one of the current research hotspots. Due to its adaptability, fault tolerance, strong learning ability, highly parallel processing ability, and artificial intelligence-based associative memory ability, many classical network models can be used effectively. It is characterized by high compression ratio and fast compression speed, which can keep the characteristics of signals and images unchanged after compression, and can resist interference during the transmission process. The embedded zero-tree algorithm is one of the more effective algorithms in current wavelet coding. It analyzes the characteristics of the coefficients after wavelet transform, organizes and encodes them effectively, and improves the coding efficiency [12]. How to combine wavelet transform and neural network in the process of image compression so as to have the common advantages of wavelet and neural network at the same time, this has always been a problem that researchers are highly concerned about and devoted to research.

This paper takes the 512*∗*512 area intercepted around as the research object, and performs zero-tree ordinary wavelet coding, multi-tree-level improved wavelet coding, improved wavelet neural network, and the coding method of this paper, respectively, and compresses the compressed size, PSNR (peak signal-to-noise ratio), MSE (mean square error), and compression time are compared and analyzed. This paper uses the characteristics of DCT coefficients to detect DCT blocks with large horizontal grayscale changes as candidate text blocks, and then using spatial relationship constraints to connect the candidate blocks into candidate regions. The compression ratio, PSNR, MSE, and compression time of the improved wavelet neural network are 9.7, 22 dB, 220, and 30 s, respectively, and 14, 29.4 dB, 74.5, and 25 s using the method in this paper. Using the method of combining wavelet and neural network in this paper not only improves the compression ratio and compression time of neural network compression coding, but also achieves better compression quality. In this paper, an auxiliary combination of wavelet transform and neural network is used for coding. First, the image to be compressed is subjected to lifting wavelet decomposition, the low-frequency coefficients obtained from the image decomposition are retained, and the high-frequency coefficients are encoded by neural network vector quantization. Finally, according to the inverse transform, the restored high-frequency coefficients and the reserved low-frequency coefficients are used for image reconstruction.

## 2. Related Work

In order to achieve a higher compression ratio, the characteristics of the human visual system can be utilized. For detected image patches, small and isolated patches are removed, and large and dense text patches are concatenated into text regions. The method also makes full use of the structural information of text lines to correct the detection results, and makes full use of the structural information to track text lines in video frames based on SSD (Smallest square distance). This is because while the human visual system is the best image processing system in the world, it is far from perfect. The human visual system's attention to images is non-uniform and non-linear, and cannot perceive any changes in the image [13–15]. For example, the change caused by the quantization error of the image coefficients cannot be perceived by the human eye within a certain range. Therefore, if the coding scheme effectively utilizes some features of the human visual system, high compression ratios can be achieved.

Wu et al. [16] found that the purpose of people observing images is to obtain useful information, and in specific applications, people do not have the same degree of concern for all visual information. Therefore, removing psychovisual redundant data leads to the loss of quantitative information. Pandey et al. [17] analyzed that, in the encoder, the mapper reduces the pixel-related redundancy by transforming the input data; the quantizer reduces the psychovisual redundancy by reducing the accuracy of the mapper output; the symbol encoder assigns the most frequently occurring quantizer output value to reduce coding redundancy. To realize the existing image compression algorithm with neural network, that is, to develop some advanced algorithms into learning algorithms and establish a neural network model, such as wavelet neural network, fractal neural network, and prediction neural network. The decoder contains only two submodules. Because the quantization operation is irreversible, there is no inverse operation module for quantization in the decoder. According to the fidelity of the decoded image to the original compressed image, Li et al. [18] believe that image compression methods can be divided into two categories: the first type of compression process is reversible, that is, the compressed image can be fully recovered from it, the original image is obtained without information loss in the process of compression and decompression, which is called lossless compression; the second type of compression process is irreversible, and the original image cannot be completely recovered, and there is a certain loss of information, which is called lossy compression. Which type of compression to choose should be comprehensively considered according to the application.

Agarwal et al. [19] gave a brief introduction to the classical coding theory, and made a comprehensive analysis of the development and current situation of the compression coding theory, and classified the compression coding methods for still images. The principle, development history, and research status of wavelet transform theory and wavelet image coding technology are systematically discussed. Sadkhan [20] used the MATLAB wavelet toolbox function to realize the simulation of image compression based on wavelet transform. The researcher expounds the basic principle and development history of artificial neural network in detail, analyzes the network structure and working principle of improving wavelet neural network, and proposes improvement measures for the shortcomings of improving wavelet network. There are two main research ideas, one is to use new and higher precision technologies to implement existing compression algorithms, and the other is to seek new image compression theories, algorithms, and the corresponding implementation techniques [21–25].

## 3. Improved Wavelet Neural Network and Digital Image Processing

### 3.1. Improved Wavelet Signal Coding

The development of the improved wavelet image compression coding algorithm can be divided into three stages: prediction coding, transform coding, and wavelet coding. Among them, predictive coding is mainly represented by differential pulse coding, and transform coding is mainly represented by JPEG compression coding. Wavelet coding is mainly represented by ordinary wavelets. In fact, wavelet transform can also belong to transform coding. It is only after the wavelet base suitable for image compression is constructed that the wavelet transform has qualitative development, especially the release of the new generation of compression coding JPEG2000. It makes wavelet coding stand out in image compression. Using balanced orthogonal multi-wavelets, the signal processing does not require complex preprocessing. It only needs to divide the original signal into odd-numbered items and even-numbered items, and combine them to form an initial vector as the input vector of the multi-wavelet.

(1) $$\begin{array}{c} F\left(i,j\right)=f_{a}\left(i,j\right)+f_{b}\left(i,j\right)+f_{c}\left(i,j\right)+\cdots +f_{n}\left(i,j\right). \end{array}$$

**Figure 1:**
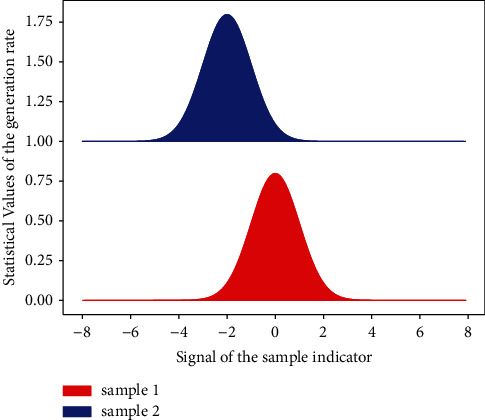
Statistical distribution of improved wavelet signal coding.

In this case, decorrelation is actually the approximation and decomposition of the two-dimensional surface formed by the image signal. The commonly used decorrelation methods are wavelet analysis or Fourier transform. To sum up, the basic principle of image compression includes the following parts: decorrelation, quantization, and direct coding. First, the image is treated as a deterministic signal, and function approximation methods such as DCT transform and wavelet transform are used to reduce the correlation of the signal, then the coefficients are quantized, the signal noise is removed, and the coefficient value is reduced. Finally, the data in Figure 1 is further removed by the method of information theory of redundancy.

Considering that the higher-frequency coefficients have less influence on the image, that is, in the wavelet coefficients, the lower-layer coefficients have a higher proportion of zeros; the author further improved the above idea. A tree is completely determined by its roots.

(2) $$\begin{array}{c} \sum_{1,1}^{i,j}\left(f\left(i,j\right)-a\left(i\right)+f\left(i-1,j-1\right)-a\left(j\right)\right)=1. \end{array}$$

That is to say, first use the method of filling rectangular blocks to find the tree root in the coefficients of the third layer, and if there is a 1 × *m* zero matrix, use it as the root to build a rectangular block. If there are nonzero coefficients in its descendants (hereafter, these nonzero coefficients are called special values), then use a special value list to record the coordinates and coefficient values of the coefficient relative to the matrix.

### 3.2. Neural Network Discrete Cosine Transform

If the binary coding scheme is adopted, assuming that the coding length of each weight of the neural network is only 2, we know that such a short coding length is difficult to guarantee the accuracy, but at this time, the coding length of a neural network individual has reached 64 × 2 = 128, according to the pattern theorem, the coding space with a chromosome bit string length of 128 contains 128 × 2 individuals, and the huge search space will cause the algorithm to converge slowly or even fail to converge at all. The above example is only for a small and medium-sized network. If it is a large network, then the length of the code string can be imagined. However, these problems can be avoided if the real number coding scheme is adopted. Therefore, this paper adopts the real number coding scheme with high search accuracy and large search space to optimize the design of feedforward neural network.

(3) $$\begin{array}{c} \frac{\int f\left(i+1,j+1\right)\text{d}i\text{d}j}{\int f\left(i,j\right)\text{d}i\text{d}j}-\frac{\int f\left(i,j\right)\text{d}i\text{d}j}{\int f\left(i+1,j+1\right)\text{d}i\text{d}j}=0. \end{array}$$

The essence of using the Huffman encoding method is to re-encode the character itself according to the statistical result, and the bit number of the obtained unit pixel is closest to the actual entropy value of the image. The process can be described by a binary tree; the characters to be encoded are represented by the leaf nodes of the tree, and each node P refers to the probability of a specific character appearing in the corresponding subtree. The two nodes with the smallest probability are selected in turn to form intermediate nodes until the root node is formed, thus completing the construction of the tree. Obviously, the root node of the final tree has a probability of 1. After completing the construction of the tree, all branches are assigned as 1 or 0, then the Huffman code of each input character in Table 1 is the sequence of digital identification on the path from the root to the leaf node.

After the image undergoes balanced orthogonal multi-wavelet transform, most of the energy of the image is concentrated on the lowest-frequency sub-image component, and other sub-image components contain less energy, so the lowest-frequency sub-image component is combined with the other sub-image components during image coding. The cosine function does not have the characteristics of local convergence. From the perspective of signal approximation, the efficiency of using the cosine function as the transformation kernel to approximate the signal is obviously not too high. Wavelet decomposition has the characteristics of fast convergence and can solve this problem very well. The signal approximation of wavelet acts locally and the approximation speed is faster than that of Fourier decomposition. It can be seen that the compression and decompression time of model 3 is short. Due to the optimization of the weights and thresholds of the improved wavelet neural network, the convergence is accelerated, and the target error is achieved in only 10 times of the improved wavelet network.

### 3.3. Improved Wavelet Performance Prediction

According to the specific application of the improved wavelet neural network, a custom threshold *T* is set. If the coefficient after wavelet decomposition is greater than or equal to the threshold *T*, it is called an important coefficient about *T*; otherwise it is called a secondary coefficient about *T*. After zero-tree coding, a small number of nodes on the quad-tree can be used to represent most of the secondary coefficients located in the high-frequency sub-bands. The zero-tree coding scanning method is performed step by step from low frequency to high frequency. The embedded code stream is formed beforehand. This structure allows the encoder and decoder to stop on any stream and ensures the best compression quality.

(4) $$\begin{array}{c} C\left(k,i,j\right)=\int k\left(i,j\right)\text{d}i\text{d}j-\int \left(k-i-j\right)\left(i,j\right)\text{d}i\text{d}j. \end{array}$$

Among them, the Huffman coding method based on wavelet transform has the highest image data code rate, the zero-search fractal image coding method has the second image data code rate, and the rectangular block. The image data code rate of the zero-tree coding method is slightly higher than that of the optimized algorithm of the rectangular block filling method, and the code rate difference between the two is only 0.01 improved wavelet *p*. However, the optimization algorithm of the rectangular block filling method needs to optimize the data after a lot of statistics, and the operation is complicated, and the rectangular block proposed in this paper. The zero-tree coding method is a compression method with similar compression performance to the optimization algorithm of the rectangular block filling method, but with simpler operation.

**Figure 2:**
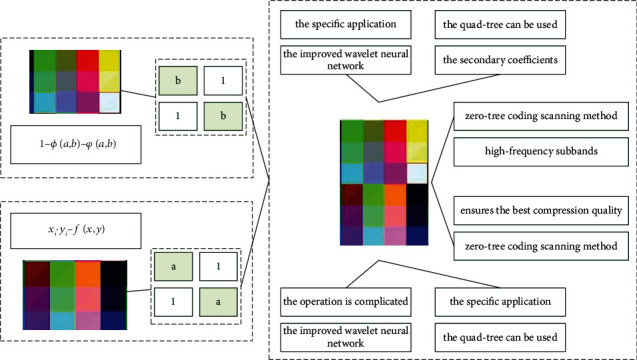
Improved wavelet coding ordering.

The encoded data sequence of Figure 2 is represented as an interval between 0 and 1, the position of which is related to the probability distribution of the input data. The longer the information, the smaller the interval the code represents, and the more bits are required to represent that interval. The continuous symbols in the information source reduce the interval according to the probability of a certain pattern, and the symbols with a high probability of occurrence have a smaller reduction range than the symbols with a small probability of occurrence, so only a few bits are added.

### 3.4. Digital Image Resolution Processing

If the digital image is subjected to DCT transform, the de-correlation effect is not good, and the calculation efficiency of DCT transform will be very slow. Considering the above problems, JPEG coding divides the image into 8 × 8 blocks before transforming, and then separates these blocks. The small blocks are independently coded, and each small block becomes an 8 × 8 real number matrix after DCT change.

(5) $$\begin{array}{c} \sum_{1,1}^{i,j}\sum_{1,1}^{i,j}a\left(i\right)\frac{f\left(i\right)}{f\left(m\right)}-\sum_{1,1}^{i,j}a\left(i\right)f\left(j\right)=\sum_{1,1}^{i,j}\frac{f\left(i\right)}{a\left(i\right)}. \end{array}$$

The upper left part of the matrix represents low frequencies and the lower right part represents high frequencies. After FDCT transformation, the coefficients are quantized according to the established coefficient quantization table. Due to the relationship of human vision, the selection of high-frequency coefficient quantization is generally more compensated than that of low-frequency coefficient selection. Coding—The encoding process of JPEG is shown in the text; decoding is its inverse process. JPEG is more efficient than DCT encoding and the algorithm is simple, so the application is very popular.

(6) $$\begin{array}{c} \left\{\begin{array}{c} 0<a\left(s,t\right)p\left(a,\left(t-b\right)\right)<a\left(s,t\right)-p\left(a,\left(t-b\right)\right),\\ a\left(s,t\right)-p\left(a,\left(t-b\right)\right)<\sqrt{a\left(s,t\right)p\left(a,\left(t-b\right)\right)}<1. \end{array}\right) \end{array}$$

When using the Mallat algorithm for signal processing, no specific wavelet function is required. When processing digital signals, it is usually assumed that the corresponding continuous function belongs to *V*0. Even so, the projection coefficient of the function in the *V*0 space is generally different from the discrete sequence obtained by sampling, but in fact, the sampled signal is directly processed as the highest resolution signal. At this time, the wavelet transform is more used as a filter. Since the signal length is fixed, in the actual application of the Mallat algorithm, the method of period expansion and reflection expansion is usually used to deal with the boundary problem, mainly to reduce the problem of slow coefficient decay caused by the discontinuity of the boundary. When using vector quantization, the size of the codebook, the image compression ratio, and the reconstruction quality should be comprehensively considered.

**Figure 3:**
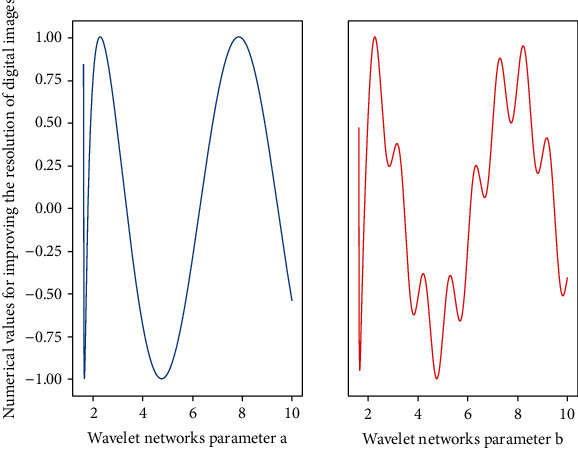
Resolution distribution of digital image with improved wavelet network. (a) Wavelet networks parameter *a*. (b) Wavelet networks parameter *b*.

We conduct experiments on both still images and video sequences of Figure 3. Images are stored in BMP format or JPEG format. We convert color image frames to grayscale images before using the algorithm proposed in this paper. The execution result of the proposed algorithm is illustrated step by step in this paper. The three-layer improved wavelet neural network algorithm is used as the classifier. In order to complementarily aggregate two sets of statistical wavelet features and a set of structured geometric features, two sets of new mixed features are constructed. Handwritten digit geometric features such as cycle number, *T*-intersection, *X*-intersection, endpoints, concave/convex, midline features, and local segmentation features were taken and decoded into 20 geometric features.

## 4. Model Construction of Digital Steganography and Image Synthesis Based on Improved Wavelet Neural Network

### 4.1. Wavelet Neural Network Data Redundancy Reduction

The luminance of the sub-bands of the detail components corresponding to the text characters of an image is different from each other. We use these differences in brightness to calculate the three eigenvalues of candidate text regions, which are used as input values to improve the training of wavelet neural network. After the neural network is well trained, new input data will produce an output value between 0 and 1. The output value corresponding to the correct text information area is completely different from the output value of the non-text area. Therefore, we can remove non-text regions using an appropriate threshold. Finally, the correct text area is extracted after processing by the dilation operation.

(7) $$\begin{array}{c} \frac{\sum_{1,1}^{i,j}a\left(i\right)b\left(i\right)c\left(i\right)}{\sum_{1,1}^{i,j}a\left(i\right)}-\frac{\sum_{1,1}^{i,j}a\left(i\right)b\left(i\right)c\left(i\right)}{\sum_{1,1}^{i,j}b\left(i\right)}-\frac{\sum_{1,1}^{i,j}a\left(i\right)b\left(i\right)c\left(i\right)}{\sum_{1,1}^{i,j}c\left(i\right)}=0. \end{array}$$

Sequential encoding is a mode that each JPEG must support, and it provides a simple and efficient image encoding scheme suitable for most applications. The enhancement system (progressive coding, layered coding) is an extension or enhancement of the basic system and the basic system must be included in the enhancement system. There is only one luminance component in a grayscale image, while a color image has one luminance component and two chrominance components. When coding a color image, each component can be coded according to the coding method for a grayscale image.

(8) $$\begin{array}{c} \int \frac{L\left(u,v\right)}{u\left(a/b\right)v\left(t^{\prime }\right)}\text{d}p\text{d}u\text{d}v=\int \frac{L^{\prime }\left(u^{\prime },v^{\prime }\right)}{u\left(\left(t-a\right)/b\right)v\left(t\right)}\text{d}u\text{d}v. \end{array}$$

After region coding and threshold coding, most of the coefficients of the transformed image are zero, and an effective method must be used to organize the nonzero coefficients and zero coefficients to ensure the maximum occurrence probability of zero consecutive coefficients with the least redundancy. In DCT image coding, zigzag scanning can be used for transform coefficients.

**Figure 4:**
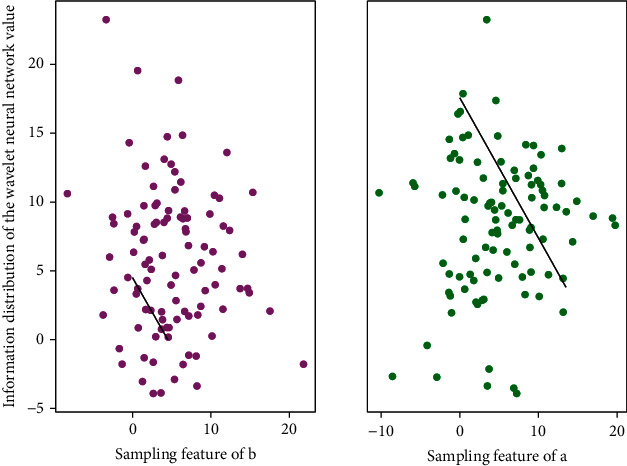
Data redundancy distribution of wavelet neural network. (a) Sampling feature of *b*. (b) Sampling feature of *a*.

First, according to the energy distribution in Figure 4 of the transform coefficients, the entire image is divided into NXN pixel blocks, and then the DCT transform is performed on the NXN pixel blocks one by one. The image coefficients with larger amplitude after transformation are mostly concentrated in the upper left corner of the image block. Compared with the other coefficients, these low-frequency coefficients include most of the content of the image, have the largest energy, and have the most important position in the transformed image, so their quantization error should be minimized. On the other hand, the high-frequency components of most images are small and have little effect on the image quality.

(9) $$\begin{array}{c} \frac{f_{u}\left(i,j-1\right)u\left(i,j\right)}{f_{u}\left(i,j\right)}-\frac{f_{u}\left(i-1,j\right)}{u\left(i^{\prime },j^{\prime }\right)}=\frac{u\left(i^{\prime },j^{\prime }\right)}{u\left(i,j\right)}. \end{array}$$

In addition, the human eye is not very sensitive to the distortion of high-frequency components, so coarser quantization can be used. Generally, the method of setting a threshold is used. The transform coefficients smaller than the threshold are set to zero, so that the code rate used to transmit the transform coefficients is much smaller than the code rate used to transmit the image pixels, thereby greatly improving the coding efficiency. Since most of the coefficients of the image are zero or close to zero after the balanced orthogonal multi-wavelet transform, the dimension of the code vector can be increased to improve the compression ratio.

### 4.2. Improved Wavelet Network Digital Steganography

The separable two-dimensional wavelet transform composed of one-dimensional wavelet and tensor product is to decompose the original image into a low-frequency signal and three high-frequency signals, and the low-frequency signal can be further decomposed into four lower-resolution sub-bands. However, how many levels of image decomposition can meet the coding requirements, although it is closely related to the complexity of the image and the length of the filter, but considering the amount of sub-band information, when a sub-band is decomposed into four sub-bands, the required four sub-bands are divided into that the entropy sum should be smaller.

(10) $$\begin{array}{c} \langle S_{\text{loc}}\left(t^{u},v\right)+S_{cls}\left(p,u\right)|S_{loc}\left(t^{u},v\right)-S_{cls}\left(p,u\right)\rangle =\langle 0|1\rangle . \end{array}$$

Under normal circumstances, the target range of ordinary images is small, there are many flat areas, the high-frequency information is relatively small, and the gray value is relatively continuous, so its autocorrelation is relatively high. However, the remote sensing image has a large area, more detailed information and richer texture than ordinary images and the continuity of gray value of pixels is also poor. The quality of the reconstructed image is good, there is basically no distortion visually, and the peak signal-to-noise ratio is also relatively high in terms of quality indicators to improve the compression ratio. Also, because the weights and thresholds of the improved wavelet neural network are optimized, the convergence is accelerated, and the target error is achieved after only 15 times of the improved wavelet network.

(11) $$\begin{array}{c} f\left(x,y\right)=\left\{\begin{array}{c} \sum_{i=0}^{n-1}x_{i}\cdot y_{i}-1,\\ \sum_{i=0}^{n-1}xy\left(x,x_{i}\right)-1. \end{array}\right. \end{array}$$

The quality of the reconstructed images is also better, but slightly worse than Model 3. In addition, it can be seen from the above table that due to the addition of wavelet preprocessing, the structure is complicated. Compared with the third model, the final compression and decompression time is longer. Therefore, during image coding, an adaptive quantization strategy can be used for sub-images and sub-image components in different directions and frequencies.

**Figure 5:**
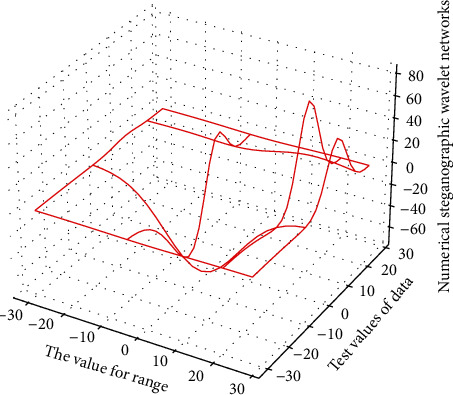
Improved wavelet network digital steganography processing distribution.

Before extracting the two-dimensional wavelet features, we perform the edge enhancement operation in Figure 5 to make up for the lack of direction selectivity. After Kirsch directional selection operation, four directional feature matrices, two endpoint matrices, ∼global digital image matrix, and 20 geometric features are combined to form a hybrid feature set I. As for the extraction of complex wavelet features, the advantages of direction selectivity and shift invariance with respect to the input image are beneficial to enhance its recognition ability; we extract 160 complex wavelet features and 20 geometric features to form Feature Set II. The experimental results show that the mixed feature set I has a higher recognition rate. The reason is that the hybrid feature set I not only extracts the orientation information of handwritten digital images, but also preserves its global and endpoint information. However, if we directly apply the two-dimensional wavelet transform to a handwritten digital image to extract wavelet features, the recognition rate will be greatly reduced.

### 4.3. Image Synthesis Compression Expansion and Noise Removal

If the coefficient of an image synthesis compression node is quantized to 0, but its sub-coefficients are not 0 after quantization, then we call this point an isolated zero, and the ordinary wavelet algorithm is an improved zero-tree based on the isolated zero. In practical applications, we will set a threshold *T* in advance according to the specific situation. If the wavelet coefficient is greater than or equal to the threshold *T*, this coefficient is called an important coefficient about *T*; otherwise it is called an unimportant coefficient about *T*. If this coefficient is an insignificant coefficient, and its sub-coefficients are also insignificant coefficients, then the coefficient is called the zero-tree root with respect to *T*.

(12) $$\begin{array}{c} \sum_{i=0}^{n-1}x_{i}\cdot y_{i}-f\left(x,y\right)x+\sum_{i=0}^{n-1}x_{i}\cdot y_{i}-u\left(x,y\right)=1. \end{array}$$

If the element value is *P* or *N*, the symbol matrix is updated, and the element value corresponding to the reconstruction table of the inverse quantizer is stored in the important coefficient matrix; if it is “*Z*”, the value of the corresponding element of the decoding matrix is reset to zero; for “*T*”, the sign matrix is updated, the corresponding value in the inverse quantizer reconstruction table is set to 0, and the sign matrix of all descendants of this point is set to “*X*”.

**Figure 6:**
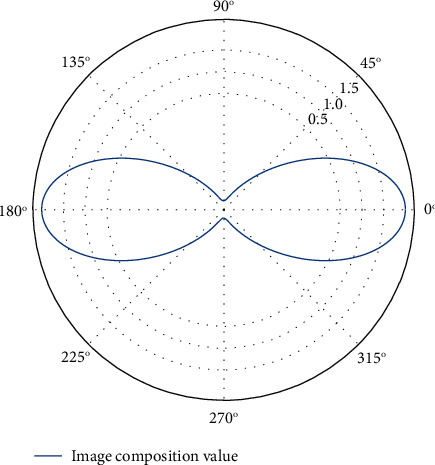
Image synthesis companding polarization distribution.

For each component of the input image in Figure 6, firstly divide it into non-overlapping 8 × 8 pixel blocks, and then perform 8 × 8 two-dimensional DCT transform from left to right and top to bottom, and the obtained “coefficients” represent the frequency components of the image block. In the 8 × 8 coefficient matrix, the one in the upper left corner is a direct current (DC) coefficient, and the other 63 are alternating current (AC) coefficients. From left to right, the horizontal frequency increases; from top to bottom, the vertical frequency is increased. The entropy obtained when a single source symbol output is observed. The maximum entropy occurs when the probability of occurrence of each symbol of the source is equal, and the source provides the maximum possible average amount of information per source symbol at this time.

## 5. Application and Analysis of Digital Steganography and Image Synthesis Model Based on Improved Wavelet Neural Network

### 5.1. Improved Weight Distribution of Wavelet Neural Network

The N L-dimensional vectors in the improved wavelet neural network are used as input samples, the number of groups *M* is divided into as the number of neurons, and network learning is carried out through corresponding algorithms, and finally the samples are divided into *M* categories according to the established rules. The more common vector quantization compression coding SOFM coding is usually used. This chapter will also use this encoding for experiments. Its average code word length is smaller than any other uniquely decodable under the premise of the same input probability set.

(13) $$\begin{array}{c} \frac{x_{i}\cdot y_{i}-f\left(x,y\right)}{M-N}-esc\left(x,y\right)=\sum_{i=0}^{n-1}\frac{x_{i}\cdot y_{i}-u\left(x,y\right)}{M-N}-esc\left(x^{\prime },y^{\prime }\right). \end{array}$$

As a result of a wavelet transform, the image is decomposed into three low-frequency sub-bands and three high-frequency sub-bands, namely, three days 1, HL1, and HH1. Because each level of processing needs to be decided twice, the size of the image after processing is reduced to 1/4 of the original, and the resolution is half of the original. In the second wavelet transform, only the sub-band is carried out, and the sub-band is further decomposed into LLI, LHl, HLl, and HHl, and the frequency range is further halved, and so on.

**Figure 7:**
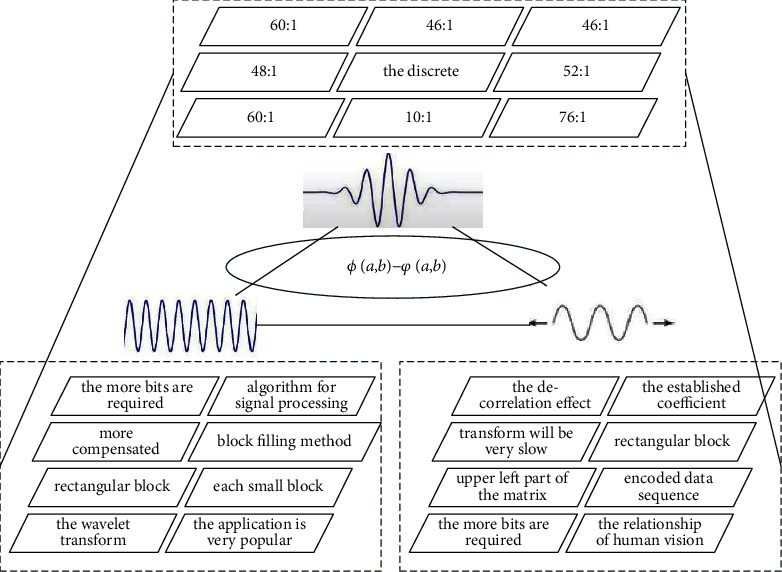
Improved wavelet neural network topology.

If the signal in Figure 7 is random, KLT is the best orthogonal transform, but since KLT has no fast algorithm, it is not suitable for image compression, and DCT is another optimal choice, using the cosine function as the transform. The kernel, which is essentially an offline Fourier transform, transfers the signal to the frequency domain after the transformation. DCT transform can be calculated by fast FFT; the difference is that the coefficients after DCT transform are real numbers.

(14) $$\begin{array}{c} \frac{\text{Psernor}\left(m,n\right)}{\log\left(m,n\right)}=10\;\log\left(\frac{mn}{\exp\left(m\right)}+\frac{m^{\prime }n^{\prime }}{\exp\left(m^{\prime }\right)}\right). \end{array}$$

The rounding operation is equivalent to making a small change to the original wavelet filter coefficients, which makes the transformation become non-linear, but does not affect the characteristics of the wavelet transform. Moreover, unlike the operation that directly quantifies and rounds the calculation results in Table 2, which causes information loss, adding a rounding step to the boosting method will not cause the original data to be unable to be accurately reconstructed, because the rounding operation does not change the original calculation.

The zero-tree coding method makes good use of the correlation between the wavelet coefficients of different layers, but does not take advantage of the correlation between the wavelet coefficients of adjacent regions in the same layer; the rectangular block filling method makes use of the adjacent wavelet coefficients in the image. The correlation between the wavelet coefficients in the region is not used, but the correlation between the wavelet coefficients in different layers is not used. The accuracy of the prediction depends on the probability distribution and correlation of the sources. In general, the predicted value and the actual value cannot be exactly the same, but they can be as close to the actual value as possible.

### 5.2. Digital Steganography and Image Synthesis Timing Simulation

The wavelet in the improved wavelet neural network adopts Daubechies wavelet. The transfer functions of the hidden layer and the output layer are, respectively, the hyperbolic tangent S-shaped transfer function and the linear transfer function. The training function of the improved wavelet network is trained according to the BFGS quasi-Newton method. The maximum number of training steps of the network is 1000 times, error is 0.001, *α* = 0.9. Taking all the pixels of an image as the input of the compression network, the scale of the network should be properly controlled, so the image is first divided.

(15) $$\begin{array}{c} \frac{\phi \left(a,b\right)-\varphi \left(a,b\right)}{\phi \left(a,b\right)+\varphi \left(a,b\right)}\frac{1}{\left|a-b\right|}=\frac{1-\phi \left(a,b\right)-\varphi \left(a,b\right)}{1+\phi \left(a,b\right)-\varphi \left(a,b\right)}. \end{array}$$

**Figure 8:**
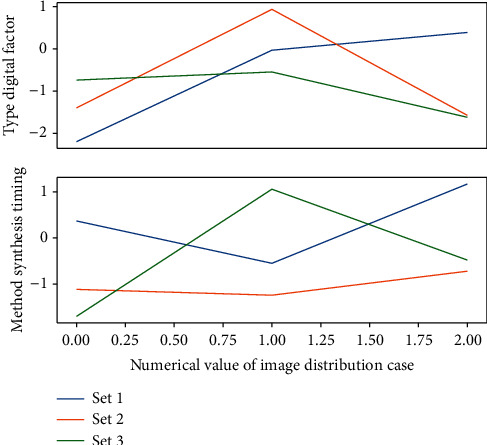
Timing distribution of digital steganography and image synthesis.

Zero continuation has a poorer effect due to the large coefficients appearing on the boundary of the signal transition; repeated boundary continuation is better than zero continuation, but the transition of the signal is still not smooth enough; the method of periodic continuation is equivalent to cyclic volume. However, if the values of the first sample point and the last sample point of the input signal are different, it will also cause discontinuities at the boundary of the signal, resulting in an increase in the local wavelet coefficients. A better way is to use symmetric period extension, which can solve the problem of discontinuous points at the boundary, but after extension, the period of the signal in Figure 8 becomes twice the original signal.

The high-frequency part after adaptive decomposition has more points with gray value of 0, which further proves that adaptive boosting has more advantages. *g* is the difference map between the original image and the reconstructed image, the gray value of all points is 0, and 100 is added to the gray value of each point; we can further calculate that the peak signal-to-noise ratio (PSNR) is infinite, the complete reconstruction of integer transformation is verified, which can make image compression truly lossless. In fact, the wavelet transform only provides a good image representation for image compression without reducing the total amount of data.

(16) $$\begin{array}{c} \frac{\iint \phi \left(a,b\right)\text{d}a\text{d}b-\iint y\left(a,b\right)\text{d}a\text{d}b}{\iint \phi \left(a,b\right)\text{d}a\text{d}b+\iint y\left(a,b\right)\text{d}a\text{d}b}⟶\text{liemitr}\left(a,b\right),\text{if}\left(a+b<1\right) \end{array}$$

When considering the frequency bands of wavelet images from the perspective of multi-resolution analysis, these frequency bands are not completely independent. Especially for each high-frequency frequency band, since they are the same edge, contour, and texture information of the image in different directions, different scales, and different resolutions from fine to coarse description, there must be a certain relationship between them. In addition, there is also a correspondence between the edge of the low-frequency band and the edge contained in the high-frequency band at the same scale.

### 5.3. Example Application and Analysis

The remote sensing image selected in this chapter is 512*∗*512. By performing different compression and coding processing on the same image, the compression quality is mainly evaluated by objective evaluation indicators such as compression ratio, compression time, PSNR, MSE, and subjective visual comparison. From the comparison of experimental data, it can be seen that the compression quality is similar when the compression ratio is larger, and the compression time is slightly higher than that of the ordinary wavelet coding. The core idea of the improved wavelet algorithm is similar to the ordinary wavelet algorithm, but it can be seen from the specific implementation that the improved wavelet is an optimization of the ordinary wavelet algorithm.

(17) $$\begin{array}{c} \sum_{i=0}^{n-1}a_{i}-b\sum_{i=0}^{n-1}f\left(x,x_{i}\right)+c\sum_{i=0}^{n-1}f_{i}\left(x-y\right)\cdot y_{i}+f\left(a,b,c\right)=1. \end{array}$$

In order to achieve the purpose of high compression ratio, the transformed coefficients should also be analyzed and processed, and appropriate quantization and coding should be carried out. For each type of handwritten digit, the output layer consists of 10 neurons. First, the Nguyen–Widrow initialization scheme 61 is used to assign values to the ownership value and the partial positive value, and then the gradient descent improved wavelet algorithm is used to update their values. We use the *J*-cut sigmoid function as the transformation function to convert the entire real value into the interval [−l, 1]. When given an image corresponding to the *i*th handwritten digit, the expected *i*th output value is +1, and the other output values are −1.

**Figure 9:**
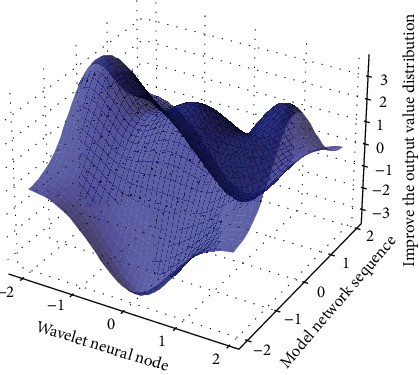
Image output value distribution of improved wavelet neural network.

The computer used for the algorithm simulation in this paper is configured as Intel(R) Pentium(R) Dual CPU T2310 processor, the CPU speed is 1.46 GHz, and the memory is 1.00 GB. The images used in Figure 9 are all grayscale images in standard tif format. Train the network with the given algorithm, then simulate, if the hidden layer neurons are K, the hidden layer of the network generates a matrix of *K* × 4096, a weight matrix of 16 × K is generated between the hidden layer and the output layer, and the output layer generates a 16 1 × threshold vector, respectively. At the same time, the high-frequency coefficients on the low-level resolution layer have less influence on the visual effect than the high-frequency coefficients on the high-level decomposition layer. Entropy coding is performed on the simulation results, and then each column vector is transformed into an image block, and all sub-images are combined into a complete image, thereby completing the image reconstruction. Therefore, during encoding, coarse quantization can be adopted for the low-level area, and fine quantization can be performed for the high-level area.

## 6. Conclusion

Aiming at some shortcomings of ordinary wavelet algorithm, the paper presents an algorithm based on improved wavelet neural network. This paper first introduces the basic principles of image data compression and several common image compression coding methods, and compresses the advantages and disadvantages of the zero-tree coding method and the rectangular block filling coding method. Secondly, the method of constructing reversible integer wavelet transform with lifting method is analyzed and discussed; a new adaptive integer wavelet lifting algorithm is given, and an adaptive lifting structure with complete reconstruction function is proposed, because it strictly limits the update steps. The sum of the filter coefficients is 1, making it difficult to construct integer transforms with it. In order to obtain the integer transformation, this paper generalizes it to a more general case. The algorithm treats edge points and uniform regions differently in the image, and integer transformations do not introduce round-off errors. Finally, the embedded wavelet coding algorithm based on wavelet transform is discussed in detail, and three main embedded wavelet coding algorithms are compared and analyzed: ordinary wavelet algorithm, improved wavelet algorithm, and SPECK algorithm. The verification results of Matlab simulation software show that the compression method proposed in this paper is simple in calculation and high in compression ratio. Compared with the existing wavelet zero-tree compression coding, the effect is better when the compression ratio is high. Visually, there is basically no distortion, the model improves the compression ratio, the convergence is accelerated, and the reconstructed image quality is also better. Compared with the methods based on traditional wavelet transform and zero-tree quantization, the operation is simple, the speed is fast, and the reconstructed image quality is high, and satisfactory results have been achieved.

## Figures and Tables

**Table 1:** Description of discrete transformation of neural network.

Description index	Root node name	Digital identification	Processing speed	Compression ratio
1	PSN	60 : 1	0.865	0.346
2	PNR	46 : 1	0.396	0.719
3	PSR	52 : 1	0.449	0.433
4	MSE	2 : 1	0.770	0.764
5	MSQE	76 : 1	0.431	0.426
6	MST	10 : 1	0.113	0.881
7	MSET	48 : 1	0.121	0.993

**Table 2:** Reconstruction of improved wavelet neural network algorithm.

Reconstruction of algorithm	Improved wavelet neural network text
Unlike the operation *M* − *N*	From mpl_toolkits.mplot3d import axes3d
That directly quantifies and *y*(*a*, *b*)	From mpl_toolkits.mplot3d import axes3d
Rounds the calculation results *f*_*i*_(*x* − *y*)	Import matplotlib.pyplot as plt
The rounding operation is *esc*(*x*′, *y*′)	From matplotlib import cm
Affect the characteristics *a*+*b* < 1	Ax.set_xlabel(‘*X*')
liemitr(*a*, *b*) of the wavelet transform	Ax.set_xlim(−40, 40)
Makes the transformation become non-linear	Import matplotlib.pyplot as plt
To the original wavelet filter coefficients	Ax.set_ylim(−40, 40)
Equivalent to log(*m*, *n*)	Ax.set_zlabel(‘*Z*')
Making a small change *φ*(*a*, *b*)	From mpl_toolkits.mplot3d import axes3d

## Data Availability

The data used to support the findings of this study are available from the corresponding author upon request.
